# High-performing trauma teams: frequency of behavioral markers of a shared mental model displayed by team leaders and quality of medical performance

**DOI:** 10.1186/s13049-017-0452-3

**Published:** 2017-11-10

**Authors:** Bjørn Helge Johnsen, Heidi Kristina Westli, Roar Espevik, Torben Wisborg, Guttorm Brattebø

**Affiliations:** 1 Department of Psychosocial Science, University of Bergen, Christiesgt 12, 5015 Bergen, Norway; 2 AFF, Norwegian School of Economics, Bergen, Norway; 3 The Royal Norwegian Naval Academy, Bergen, Norway; 4 Anesthesia and Critical Care Research Group, Faculty of Health Sciences, University of Tromsø, Tromsø, Norway; 5 Department of Anesthesia and Intensive Care, Haukeland University Hospital, Bergen, Norway; 6 Department of Clinical Medicine, University of Bergen, Bergen, Norway; 7 Norwegian National Advisory Unit on Trauma, Oslo University Hospital, Oslo, Norway

**Keywords:** Trauma team leader, Teamwork, shared mental model

## Abstract

**Background:**

High quality team leadership is important for the outcome of medical emergencies. However, the behavioral marker of leadership are not well defined. The present study investigated frequency of behavioral markers of shared mental models (SMM) on quality of medical management.

**Method:**

Training video recordings of 27 trauma teams simulating emergencies were analyzed according to team -leader’s frequency of shared mental model behavioral markers.

**Results:**

The results showed a positive correlation of quality of medical management with leaders sharing information without an explicit demand for the information (“push” of information) and with leaders communicating their situational awareness (SA) and demonstrating implicit supporting behavior. When separating the sample into higher versus lower performing teams, the higher performing teams had leaders who displayed a greater frequency of “push” of information and communication of SA and supportive behavior. No difference was found for the behavioral marker of team initiative, measured as bringing up suggestions to other teammembers.

**Conclusion:**

The results of this study emphasize the team leader’s role in initiating and updating a team’s shared mental model. Team leaders should also set expectations for acceptable interaction patterns (e.g., promoting information exchange) and create a team climate that encourages behaviors, such as mutual performance monitoring, backup behavior, and adaptability to enhance SMM.

## Background

In health care, leadership is an important factor in patient outcome. In recognition of this importance, knowledge about medical leadership is stated as a requirement for healthcare personnel by the UK General Medical Council [1]. Furthermore, the Academy of Medical Royal Colleges and UK National Health Service published the Medical Leadership Competency Framework [2], which was developed based on the concept of shared leadership. Shared leadership is not restricted to people who hold designated leadership roles but comprises a shared sense of responsibility amongst several people for the success of the organization and its services. There is a consensus about the importance of leadership, especially of team leadership [3]; however, a clarification is still needed of the specific elements in optimal medical leadership skills. For instance, many types of life-support courses neglect the non-technical skills (NTS) needed for increased medical effectiveness [4]. As a result, Flin and Maran [4] outlined an NTS framework for training medical teams. In one study of team training in emergency medicine, the specific leadership skills of medical team leaders were observed [5]. The results suggested that leadership was a critical factor in team performance, and the researchers argued that leadership could be seen as an integral component in centralizing communication, facilitating coordination, allocating resources, and keeping an accurate shared mental model (SMM) of the evolving scenario as a whole [5].

A promising theoretical framework in the study of team behavior is the so-called SMM approach [6]. From a series of studies conducted on military tactical expert teams, these authors found that effective team performance under a high workload depended on team members’ ability to apply a shared understanding of the task, the structure of the team, and each team member’s role within it. This proposed beneficial cognitive construct was referred to as a SMM [6] and is assumed to enable individual team members to predict task needs and the intended actions of other members. The construct serves as an immediate and internalized understanding of how team members coordinate behavior and choose different actions, without explicit demands being made for such coordination. In a simulated trauma team-training scenario, the SMM approach was compared to the NTS behavioral marking system [7]. The results showed that both approaches were useful in predicting quality of medical performance, but in a regression model, the SMM approach predicted treatment outcome even when controlling for the NTS markers. However, Westli et al. [7] investigated the behavioral indicators of SMM for complete teams rather than for the team leaders; thus, the specific behavioral characteristics of the team leaders of high-performing medical teams still need to be characterized.

Team leadership is defined as “the team leader’s ability to direct and coordinate the activities of other team members, assess team performance, assign tasks, develop team knowledge, skills, and abilities, motivate team members, plan and organize, and establish a positive atmosphere” [3]*.* On the other hand, if team leaders fail to guide and structure the team members in coordinating and adapting their actions, the result might be poor and ineffective team performance [8].

Team leadership thus positively influences team effectiveness by facilitating problem-solving abilities through cognitive processes (e.g., SMM), coordination processes, and collective motivation and behaviors (e.g., performance expectations) [9, 10]. Salas et al. [11] described five categories of teamwork behavior that characterizes well-functioning teams: leadership, team orientation, back-up behavior, monitoring, and adaptability (Table 1). The mechanisms that connect these teamwork behaviors are SMM, trust, and closed-loop communications. The team leader has a role in the creation, maintenance, and accuracy of the team’s SMM, which information sharing could facilitate. When SMMs are present in the team, information is shared without an explicit demand for relevant information (i.e., “push” of information). Furthermore, team leaders create situational awareness (SA) by, for example, updating status and intentions, and create a team climate (e.g., team initiative) that encourages behaviors such as mutual performance monitoring, supportive behavior, and an adaptability to enhance SMM.

**Table 1:** Team leader behavioral markers of Shared Mental Models based on the categories described by Salas et al. [11] and its relation to the observed variabes in the present study

Shared Mental Model categories	Behavioral markers	Observed behavior
Team orientation	Share information	Provide information *before* being asked (“push” of information)
Backup behavior	Provide support	Provides assistance *before* being asked
Monitoring and adaptability	Team initiative	Provides guidance or makes suggestions to other team members
Leadership	Communicating situational awareness	Providing updates to the team

Based on the theoretical framework of SMM, the first aim of this study was to investigate whether behavioral markers of SMM in team leaders are associated with subject matter expert (SME) rating of team performance. We hypothesized that all behavioral markers of SMM would be positively correlated with external ratings of medical team performance. The second aim of the study was to investigate whether the higher performing teams were characterized by more frequent behavioral markers of SMM demonstrated by team leaders compared to lower performing teams. We hypothesized that higher performing teams would show higher frequencies of all behavioral markers of SMM.

## Method

### Participants

Video recordings of 27 trauma-team leaders training in simulated emergency situations (18 males and 9 females) were used. The recordings were included based on their audio and video quality, type of injury treated (identical scenarios), and hospital category. The last was assessed to ensure an even distribution of hospital size.

### Research settings

The present study is a re-analysis of the Westli et al. [7] study, focusing only on team leader behavioral markers of SMM. The videos were recorded as part of a nationwide training program (BEST: Better & Systematic Team Training (former Better & Systematic Traumacare), which introduced a systematic approach to training trauma teams [12].

The simulation consisted of two scenarios. The first case involves an adult pedestrian hit by a car. The patient is unconscious at arrival but has adequate ventilation and circulatory status. The radiographic examinations reveal a small pneumothorax and a stable pelvic fracture. The team was expected to decide on a CT scanning of the patient’s head after having performed a regular trauma assessment.

The second case involves a young construction worker who has fallen 6 meter from a scaffold. He is awake but has obvious clinical signs of circulatory insufficiency, abdominal pain, and a pelvic fracture. The radiographic examinations show an apparently normal chest but an open-book fracture of the pelvis. He is in need of volume/blood replacement and immediate surgery.

The trauma teams involved in the simulated scenario consisted of six or seven medical professionals. Each team comprised a surgeon (team leader), an anesthesiologist, a nurse anesthetist, two emergency room nurses, a lab technician, and a radiographer. The simulations were organized and took place in the trauma room in the teams’ own hospitals. Each hospital’s own team set-up, procedures, and equipment were to be followed, and team members acted out their own professional roles in the scenarios, thus increasing the ecological validity of the study. The same simulation scenarios were used for all teams and were based on real patient cases; however, behavioral markers of SMMs were recorded only for the team leaders.

The teams were expected to have the necessary knowledge and skills to perform ABCDE procedures known from ATLS [13], suitable for assessing a trauma patient. The teams’ medical management in the videos was observed in ordet to to assess the quality of the medical performance. Two experienced medical clinicians independently scored the video recordings to estimate the medical quality based on an a priori set of performance criteria. During the simulation, the teams were to ascertain the patient’s status by assessing the following: Airways, Breathing, Circulation and hemorrhage control, Disability, and Environment and exposure. Based on these observations, the team was to decide to transfer the patient to either CT or surgery. The clinicians (SMEs) were selected based on their medical expertise and personal experience in trauma care. Accordingly, they were familiar with the procedures expected to be performed, as well as with the two trauma simulation scenarios. They received rater training to establish a common reference framework for evaluating each of the targeted performance criteria.

### Data collection

The quantitative analyses were performed using the Noldus Observer XT software system, which enables observable behavior to be scored and analyzed [14]. An accumulative (moment-to-moment) quantitative approach was used, in contrast to a single global assessment of team performance.

These variables are based on the Anti-Air Teamwork Observation Measure (ATOM) [15] and measured as number of behavioral indicators per minute. The behavior measured by ATOM and its relation to team-leader behavioral markers of SMM are presented in Table 1. Three psychologists trained in observing and rating the frequency of SMM indicators independently scored the video recordings in random order.

A global measure of medical management (quality of medical performance) was performed by two independent SMEs in trauma. Medical management measured only the technical skills exhibited by the trauma teams and was rated on an ordinal 5-point scale from 1 (poor) to 5 (good).

Two groups were formed based on the median split on the medical management variable. Teams scoring on the median (median = 4) were included in the higher performing group; thus, 9 team leaders were categorized in the lower performing group and 18 were assigned to the better performing group.

### Data analyses

Since several of the variables showed a positive skewed distribution, square root transformation was applied in the analyses. Intra class correlations (ICC) were used as measures of inter-rater reliability. The ICCs were calculated using IBM SPSS Statistics 23. When analyzing inter-rater reliability of team-leaders the average ICC was reported using a two-way random design and consistency type. When calculating the inter-rater reliability of medical management a one–way random model and was used. Other correlational and differences between means were analyzed using Statistica version 13.2. Correlations between behavioral markers of SMM and SMEs ratings of medical management were investigated by means of Pearson’s product moment correlations (one-tailed tests are marked). An independent group design was used to test for differences in indicators of SMM offered by team leaders between the two groups. These differences were investigated using separate *t*-tests for each behavioral marker. A *p*-value of < 0.05 was considered statistically significant. Effect sizes are based on Cohens *d*.

## Result

Inter-rater reliability for the scoring of indicators of team-leaders SMM was ICC = .69 (95% confidence interval between .33 and .91). The inter-rater reliability for scoring of Medical Management was ICC = .75 (95% confidence interval between .55 and .87).

The correlational analyses revealed a positive correlation between leaders sharing information without an explicit demand for the information (“push” of information) and quality of medical management (*r* (27) = .483, *p* < .011). A positive correlations were also found for the relation between supporting behavior acted by the team leader (support without demand for it), as well as updating/communicating SA and quality of medical management (*r*(27) = .353 *p* < 0.035 (one-tailed) and *r*(27) = 0. 437, *p* < 0.030, respectively). No correlation was found between guidance/suggestions from the team-leader and the Medical Management variable (see Table 2 for an overview of all intercorrelations).

**Table 2:** Inter-correlations between team leaders’ push of information, updates, supporting behavior, and Guidance/suggestions and the subject matter experts’ evaluation of quality of medical management of the cases (*N* = 27)

	1	2	3	4
1. Push of info				
2. Updates	.68**			
3. Support	.63**	.6**		
4. Guidance/suggestions	−.20	−.55**	−.49**	
5. Medical management	.48**	.42*	.35*a*	0.40*

* = *p* < .05** = *p* < .001

a = one-tailed test

**Fig. 1:**
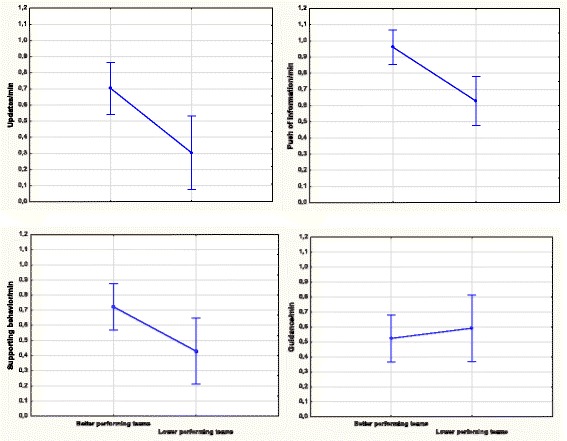
Frequency of updatings (upper left panel), "push" of informations (upper right panel), support (lower left panel) and suggestions and guidance (lower right panel). The data are square-root transformed and presented as observed behavior per minute and also separated for higher and lower performing teams

When separated into two groups based on their performance scores, team leaders differed for the behavioral marker of information sharing; leaders of better performing teams showed higher rates of push of information (*t*(25) = 3.371, *p* < 0.001; Effect size *d* = 0.414). Leaders of the better performing teams also revealed a higher frequency of updating/communicating SA (*t*(27) = 2.957, *p* < 0.006; Effect size, *d* = 1.27) compared to the poorer performing teams. Furthermore, better performing teams had team leaders characterized by higher numbers of supporting behavior without an expressed demand for support (*t*(27) = 2.265, *p* < 0.032; Effect size *d* = 1.03). No difference was found for the behavioral marker of guidance/suggestions, *t*(27) = .0516, *p* < .610; Effect size *d* = 0.01 (see Fig. 1 for an overview of between-groups results).

## Discussion

In the present study, all behavior indicators of SMM were positively correlated with SME evaluation of the quality of medical performance. Furthermore, compared to lower performing teams, leaders of better performing trauma teams demonstrated more information exchange without an explicit demand for it, more updating, and more supporting behavior.

The positive association between team leaders’ behavioral markers of SMM and external raters of the quality of the medical treatment indicates that SMM may be a relevant framework for studying and training trauma teams. This result is in line with previous research showing the impact of NTS on the outcome of medical performance [16, 17]. The significant associations between observed behavior and performance were in the medium range [18]. However, this paper expands previous knowledge by emphasizing the role of trauma team leadership.

When separating the subjects into better and lower performing teams, team leaders in the better performing group showed more frequent behavioral markers of SMM on three out of four variables. The better performing teams had leaders displaying the SMM category of leadership by updating their team more often. In this way, the leaders initiated a shared SA and mutual understanding, which enabled the team to synchronize their efforts. We also suggest that team leaders who more frequently updated their team helped their team to be on track by maintaining the existing SMM, or if necessary, changing the team’s SMM. Thus, these results follow the theory of SMM, in which teams with the best SMM can coordinate, communicate, and eventually perform better than teams with a less sophisticated understanding of the actual situation the team faces. Research has accumulated substantial support for the general presumption that SMMs are associated with better team effectiveness (see overview [19]).

The better performing teams were also characterized by leaders displaying the SMM category of team orientation. These leaders offered information without explicit demands to a greater extent than the lower performing teams. Hence, to coordinate their activity, teams with SMM will change their communication patterns from pulling (requesting) to pushing (presenting) information when the workload increases. This finding is in line with our previous work, in which we found that military expert teams with SMMs changed their communication style, showing an increase in “push” of information from low- to high-intensity situations [20]. Teams without SMM did not change their patterns of information transfer. According to Entin and Serfaty [21], this shift in communication pattern is reflected in the ratio of transfers of information divided by requests for information (“the global anticipation ratio”). An increase in “the global anticipation ratio” during high workload is seen as a strong indication of SMM [21]. Orasanu [22] found that superior teams increased the push of information from team members and reduced requests for information from the team leader during high workload periods.

In our study, better performing teams also had team leaders who to a larger extent were involved in supportive behaviors without explicit demands from the team members. The presence of both “push” of information and the offer of implicit supportive behavior without explicit demands could be seen as an indicator of the presence of an evolved SMM. The results revealed large effect sizes for updating and support behavior, and a small effect for “push” of information [18]. Contrary to our hypothesis, monitoring/adaptability did not separate leaders of better and lower performing teams. These variables comprise leaders providing guidance or making suggestions to other team members. It could be argued that suggestions or guidance are less relevant because trauma teams consist of individuals with clearly pre-defined and specialized roles. Thus, team leaders’ guidance or suggestions would not be as useful or might even interfere with the team members’ role behavior.

The present study could be viewed in light of a lead-by-example strategy. It is possible that better performing teams were encouraged by their leader to display more SMM behavior themselves, thereby enforcing better SA within the group. Hence, a better process, including the SMM mindset, enabled them to perform better collectively. Teams that obtain a collective team leadership process have an increased capacity for mastering fluctuating and complex situations.

### Limitations of the study

Some limitations of the study should be mentioned. First, ICC in small groups tend to be higher than larger groups. This could result in an inflated inter-rater reliability. However, the magnitude of the reliability coefficient could suggest that the inter-rater reliability was acceptable even with the small number of raters. Second, allocation to better or lower performing groups was based on the performance of local teams drawn from their respective hospital. This could result in a restricted range due to local culture and other influences that create teams more alike. However, the strong side of using local teams is an increased generalization of the effects observed. The third limitation of the study, could be the lack of control of confounding variables. Examples being level of team-member's expertise, verbal fluency and possible conflicts within the teams.

## Conclusion

The results of this study indicate that the team leader is important as the initiator in generating and updating a team’s SMM. Furthermore, they show that team leaders need to set expectations for acceptable interaction patterns (e.g., promoting information exchange) and create a team climate that encourages behaviors, such as mutual performance monitoring, backup behavior, and adaptability to enhance SMM. This need applies especially when stress levels are high.

We argue, as have others (e.g., [10, 22]), that team leaders ultimately facilitate and determine team effectiveness, not only by synchronizing and combining the individual contributions of each of the team members but also by ensuring that team individuals understand their interdependence and value, in addition to the obvious benefits of working together in challenging situations. Even though the team leaders are responsible for initiating and maintaining SMM, we advocate that in an ideal world, as many team members as possible contribute to and secure reinforcement of the needed teamwork processes.
